# Safety, efficacy and immunogenicity evaluation of the SAG2 oral rabies vaccine in Formosan ferret badgers

**DOI:** 10.1371/journal.pone.0184831

**Published:** 2017-10-04

**Authors:** Ai-Ping Hsu, Chun-Hsien Tseng, Jacques Barrat, Shu-Hwae Lee, Yu-Hua Shih, Marine Wasniewski, Philippe Mähl, Chia-Chia Chang, Chun-Ta Lin, Re-Shang Chen, Wen-Jane Tu, Florence Cliquet, Hsiang-Jung Tsai

**Affiliations:** 1 Division of Biologics, Animal Health Research Institute, Council of Agriculture, New Taipei City, Taiwan; 2 Graduate Institute of Veterinary Medicine, School of Veterinary Medicine, National Taiwan University, Taipei, Taiwan; 3 Nancy OIE/WHO/EU Laboratory for Rabies and Wildlife, French Agency for Food, Environmental and Occupational Health & Safety, Technopôle agricole et vétérinaire, Domaine de Pixérécourt, Malzéville, France; 4 Animal Drugs Inspection Branch, Animal Health Research Institute, Council of Agriculture, Miaoli County, Taiwan; 5 VIRBAC Laboratories, Carros Cedex, France; 6 Zoonoses Research Centre, School of Veterinary Medicine, National Taiwan University, Taipei, Taiwan; Instituto Butantan, BRAZIL

## Abstract

Since 2013, rabies cases have been reported among Formosan ferret badgers in Taiwan, and they have been shown to be the major reservoirs for Taiwanese enzootics. To control and eradicate rabies, the authorities plan to implement a vaccination programme. Before distributing live vaccines in the field, this study assessed the safety, efficacy, and immunogenicity of SAG2 vaccine on ferret badgers by direct oral instillation. After application of 10^9^ TCID_50_/dose, no virus was excreted into the oral cavity 1–7 days post-application, and safety was also satisfactorily verified over a 266-day period. Moreover, despite the low level of rabies virus neutralising antibodies induced after vaccination of a 10^8^ TCID_50_/dose, the efficacy assessment revealed a 100% survival rate (15/15) of vaccinees and an 87.5% fatality rate (7/8) in control animals after a challenge on the 198th day post-vaccination. The immunisation and protection rates obtained more than 6 months after a single vaccination dose demonstrated that SAG2 is an ideal vaccine candidate to protect Formosan ferret badgers against rabies in Taiwan.

## Introduction

Rabies is an important zoonotic disease, and canine rabies is still considered a major concern in Asia, Africa and Latin America [1–4]. In most developed countries, canine rabies has been eliminated through parenteral vaccination campaigns of dogs and strict prophylactic measures [5], but rabies is maintained in diverse wildlife species (e.g. foxes, raccoons, wolves and skunks) [6, 7].

Taiwan, a geographically isolated island, had been considered rabies-free since 1961 [8, 9], with just three human cases imported from China or south-eastern Asia [10, 11]. In 2013 however, rabies was discovered in Formosan ferret badgers (*Melogale moschata subaurantiaca*) [9]. In all 6,626 animals were tested from 2013 till the end of 2016, including 3,781 dogs, 1,416 ferret badgers, 384 gem-faced civets (*Paguma larvata taivana*), 496 other wildlife species, 382 bats and 167 cats. The epidemiology surveys indicated that Formosan ferret badgers were the major carnivores involved, with an occurrence rate of over 98.57% (553/561) in all the positive cases diagnosed. They were followed by gem-faced civets with 1.07% (6/561), and 0.18% (1/561) each for a dog and an Asian house shrew (*Suncus murinus*) [12]. These data suggest that the ferret badger could be the rabies reservoir in Taiwan.

Rabies in ferret badgers has been also evidenced in mainland China and is even a considerable threat to public health. Around 3,000 human rabies deaths are caused each year in China by rabid dogs (>95%) [2]. In some districts of Zhejiang province in south-eastern China, 80% of human rabies cases were attributed from 1994 to 2004 to Chinese ferret badgers (*Melogale moschata*) [13]. In Taiwan, epidemiology surveys showed Taiwan ferret badger rabies virus (RABV-TWFB) affected Formosan ferret badgers restrictively, and there have been no indigenous human cases caused by RABV-TWFB since the outbreak in 2013 [11]. Cases affecting gem-faced civets remain sporadic, but the enzootic situation in ferret badgers might lead to spillover events, enabling new reservoir species to emerge, and subsequently complicating rabies management.

In recent decades, oral rabies vaccination programmes have been undertaken in Europe (since the late 1970s) and North America (since the 1990s). These have contributed to successful rabies control among wildlife, with the elimination of fox rabies in Western, Central and Northern Europe [14–16]. One of the oral vaccines used is SAG2 (SAD Avirulent Gif), a modified live avirulent rabies virus strain developed from the SAD Bern virus through a two-step mutation selection with monoclonal antibodies [17]. Indeed, SAG2 is one of the two vaccines recommended by World Health Organization (WHO) for oral vaccination [18, 19]. This vaccine has been closely investigated, particularly in order to assess its efficacy among various carnivores such as foxes, raccoon dogs, jackals, raccoons, skunks, coyotes, and dogs, and to explore safety issues concerning target and non-target animals in both experimental and field conditions [20–26]. Furthermore, after distribution of more than 20 million SAG2 baits throughout Europe, no vaccine-induced rabies has ever been detected in areas vaccinated with this oral vaccine [16].

In order to achieve the “One World, One Health” goal, the Taiwanese government considers epizootic and zoonotic management a major objective. To eliminate ferret badger rabies from Taiwan, oral vaccination is one of the tools considered for use in a future control programme. Before releasing live vaccines in the field, a thorough evaluation of the vaccine’s efficacy and safety under controlled conditions is first necessary. The purpose of this study was therefore to assess the feasibility of oral vaccination in Taiwan under controlled conditions by investigating the safety, immunogenicity and efficacy of the SAG2 vaccine on ferret badgers.

## Materials and methods

### Vaccine virus and challenge virus

The SAG2 vaccine (RABIGEN^®^, Virbac Laboratories, Carros, France) is a modified live rabies vaccine [15]. The tested vaccine consisted of two SAG2 suspensions (referenced as TAIWAN-4NPC), one corresponding to the vaccine, with a titre similar to that used in a vaccine bait (i.e. 10^8^ TCID_50_/dose), and the other containing a ten-fold higher titre than the maximum found in a vaccine bait. The products arrived in the laboratory frozen and were stored at -20°C until use. Before the SAG2 vaccines were used on the animals, virus titres were determined by titration procedures on BSR cells as previously described [27].

The challenge rabies virus was prepared with a homogenate mixture of sub-maxillary salivary glands from 24 naturally rabid ferret badgers obtained from the field and found to be infected by RABV-TWFB of genetic group II. Before mixing the homogenate, all the viruses were sequenced at gene G, and nucleotide sequence identities equal to or greater than 99% were verified within the selected virus homogenate.

### Animal ethics and husbandry

The use of Formosan ferret badgers was approved by the Institutional Animal Care and Use Committee (IACUC) of the Animal Health Research Institute, Taiwan (Permit No. IACUC# A05005) and the IACUC of the Animal Drugs Inspection Branch of AHRI, Taiwan (Permit No. IACUC# 103-B03). Furthermore, all animal experiments complied with the Animal Research: Reporting of In Vivo Experiments guidelines and national regulations for the use of experimental animals (Management Regulations for Laboratory Animal Care and Use Committee or Group Setting; published in 2001 and revised in 2013). All experimental procedures (oral administration of vaccine, blood collection, saliva swab collection, and challenge) were performed under isoflurane anaesthesia at 1% to 4% by inhalation, and all efforts were made to minimise suffering.

Before rabies virus inoculation, the ferret badgers were kept in rooms made of smooth metal meshwork (at 5 badgers per room). After inoculation, isolating individual animals from each other was necessary for preventing transmission through biting; thus, each ferret badger was maintained in an isolated stainless steel cage (55.5 cm × 45 cm × 31.5 cm). The housing environment for the ferret badgers was equipped with wood for climbing and plastic boxes for burrowing and hiding. Commercial dog feed, and pork, beef, or boiled eggs were provided to ferret badgers daily. The animal facility staff verified the ferret badgers’ activity and cleaned their cages twice daily. No adverse events other than rabies-related signs developed. The animal facility staff was appropriately trained to recognise the clinical signs caused by rabies. To prevent animal suffering, at the onset of clinical signs indicating rabies, the animals were humanely euthanized with isoflurane overdose (isoflurane at 5%) until one minute after the heart rate and breathing stopped.

### Animals used and group allocation

Thirty-three Formosan ferret badgers (20 females and 13 males) were used in this study. All the ferret badgers, who weighed approximately 1 kg and were of a similar age (judged according to the level of teeth wear), were captured in Miaoli County, Taiwan (a rabies-free zone), were tagged with microchips for individual identification, and were isolated for more than 6 months as a quarantine observation period before being vaccinated against rabies. Before vaccine administration, animals were randomly allocated into three groups: Group 1 (n = 10) was assigned as the safety test group to receive 10 doses of SAG2 (10^9^ TCID_50_/animal), Group 2 (n = 15) was assigned as the efficacy test group to receive 1 dose of SAG2 (10^8^ TCID_50_/animal), and Group 3 (n = 8) was assigned as the control group to receive sterile PBS.

### Vaccine administration virus and animal challenge

Food was withdrawn from the animals before vaccination (D-1). On Day0 (D0), animals were slightly anaesthetised with isoflurane, and then either 1 ml of SAG2 with 10^9^ TCID_50_, SAG2 with 10^8^ TCID_50_ or PBS was administered to each animal in Groups 1, 2 and 3 respectively by instillation in the oral cavity via 3-ml syringes connected to feeding cannulas.

On the day of the challenge (D198), the rabies virus was thawed out and then diluted to 10^3.5^ mouse intracranial lethal dose 50% (MICLD_50_) per ml. Before virus inoculation, ferret badgers from Groups 2 and 3 were anaesthetised using isoflurane at 1%-4% by inhalation, and then challenged intramuscularly (masseter muscle) with 0.5 ml of the challenge virus suspension, corresponding to 10^3.2^ MICLD_50_ (equivalent to 50 ferret badger LD_100_). After the challenge, the challenge virus dose was further confirmed by back-titration in BALB/c mice (aged 3–4 weeks) inoculated by intracranial injection to verify the MICLD_50_ titre..

### Clinical observation

Ferret badgers from Group 1 were observed daily and these observations recorded from D1 to D266 to assess whether there were any side-effects or signs of rabies caused by SAG2 application. They were then humanely euthanised with isoflurane overdose for post mortem examination. Ferret badgers from Groups 2 and 3 were observed daily from D1 to D378, i.e. over a period of 180 days post-challenge to record clinical manifestations, and then subjected to euthanasia, post mortem examination and rabies diagnosis.

### Rabies virus neutralising antibodies

Blood samples from all the animals were collected at the jugular vein on D-2 (two days prior to the vaccination), D14 (14 days post-vaccination), D30, D45, D60, D90, D120, D150, and D180 to measure the virus neutralising antibody (VNA) titres. Additional blood samples were collected from Groups 2 and 3 on D198 and D205 (7 days post-challenge) to check the anamnestic response, and again at the end of the efficacy study (D378). Additional blood was also sampled from Group 1 on D266 (at the end of the observation period). After the formation of a blood clot and centrifugation, serum samples were collected and stored at -20°C until analysis based on the fluorescent antibody virus neutralisation (FAVN) test according to the technique described by Cliquet et al. [28] with a positivity threshold of 0.5 IU/ml.

### Examination of SAG2 virus in saliva

According to WHO guidelines for the safety evaluation of modified live-rabies vaccines in dogs, when administered orally at 10 times the recommended dose, there should be no or minimal excretion of virus following vaccination and there should be no persistent infection by the vaccine virus [16]. Based on the above, the safety test group (Group 1) was evaluated for the potential of examination of SAG2 virus in saliva and Group 3 was used as the negative control for this virus examination test. On D-2 and from D1 to D7, saliva swab samples were taken from Groups 1 and 3 to test for the presence of the virus on susceptible cells with a rabies tissue culture infection test (RTCIT) [29] and the presence of rabies nucleoprotein RNA by real-time RT-PCR. This real-time RT-PCR assay was designed specifically to detect SAG2 virus nucleoprotein. Briefly, after sample RNA was extracted with an automatic nucleic acid extraction device (taco™; GeneReach USA) in combination with a taco preload DNA/RNA extraction kit (GeneReach USA), a one-step real-time RT-PCR was performed on a Roche LightCycler^®^ 480 II (Roche, Switzerland). The reaction mixture (based on KAPA PROBE FAST Universal One-step qRT-PCR Kit; Kapa Biosystems) of 20 μl contained 3 μl of sample RNA, 0.4 μl of KAPA RT Mix (50×), 10 μl of KAPA Probe Fast qPCR Master Mix (2×), 300 nM final concentrations of each primer (forward primer 5′-GGAGGCATGGAACTGACAAGAG -3′ and reverse primer 5′-CAGACTCAAGAGAAGACCGACTAAG -3′), and a 200 nM final concentration of probe (5′-FAM-CCCACTGTCCCTGAGCATGCGT-BHQ-1-3′) subjected to thermal cycling: 30 min at 42°C, 95°C for 5 min, 50 cycles of 95°C for 3 s and 60°C for 20 s. Fluorescent signals were collected at the end of the elongation stage, and the crossing point (Cp) for a specific RNA concentration was automatically recorded by a LightCycler^®^ 480. The analysis for each RNA sample was conducted in triplicate, and positive results were determined when all three wells showed fluorescent signals with a mean Cp for the triplicate values below 39.70.

### Post mortem examination

The surviving animals were humanely euthanised at the end of the observation periods (D266 for Group 1; D378 for both Groups 2 and 3) with isoflurane overdose. Specific brain tissues (hippocampus, hypothalamus, cerebellum and medulla oblongata) and salivary glands were collected from all animals and submitted to rabies diagnosis by the direct fluorescent antibody test (FAT) [30] and RTCIT on neuroblastoma cells [29]. Moreover, to clarify whether there was any residue of SAG2 nucleic acids resulting from possible replication in ferret badgers, the salivary glands and brain tissues from animals in Group 1 were further analysed with the real-time RT-PCR specific to SAG2 with the protocol described in section 2.6.

### Data analysis and statistics

Statistical assessments were conducted with the SAS software (version 9.4). Statistical comparisons of VNA titres for Groups 1 or 2 before the rabies challenge but excluding the pre-vaccination titres were first analysed for normality, and then the mean or median titres were compared through a one-way analysis of variance (ANOVA) or Kruskal-Wallis test with a 95% confidence interval according to the distribution of data (normal or not). In order to investigate the challenge effect on Group 2 dynamically, after the normality tests, a paired t-test or Wilcoxon signed-rank test was applied for separate comparisons. For statistically comparing the survival curves between Groups 2 and 3 after challenge, the log rank test was performed.

## Results

### Safety of SAG2 in Formosan ferret badgers

#### Clinical observation and rabies virus neutralising antibodies

**Fig 1:**
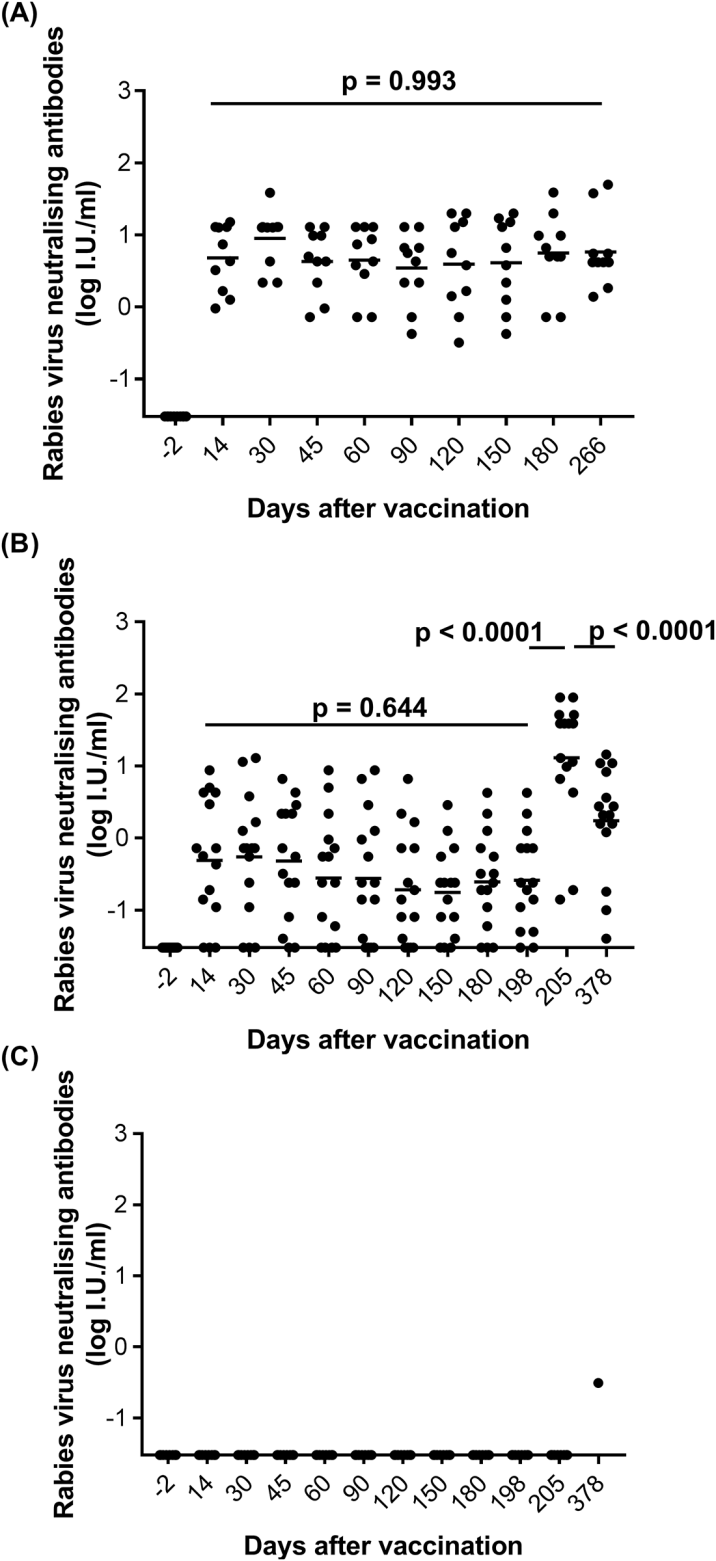
Aggregate data of rabies VNA titres (log IU/ml) for each group at each time point. Rabies VNA titres of each animal in Group 1 **(A)**, Group 2 **(B)**, and Group 3 **(C)** were respectively displayed, and the geometric means at each time point were indicated with the line in each group. The X-axis stands for the time point (days after vaccination) that animals were bleed for the FAVN test. The solid circle icons are the VNA titre value from each animal in each group at each time point (the digital data for individual animals refers to S1 Table). The statistical analysis suggested that there were no significant differences of antibody titres measured at any time point after vaccination in Group 1, and after vaccination but before challenge in Group 2; however, there were significant differences between before and l-week after challenge and also between l-week and half year after challenge in Group 2.

**Fig 2:**
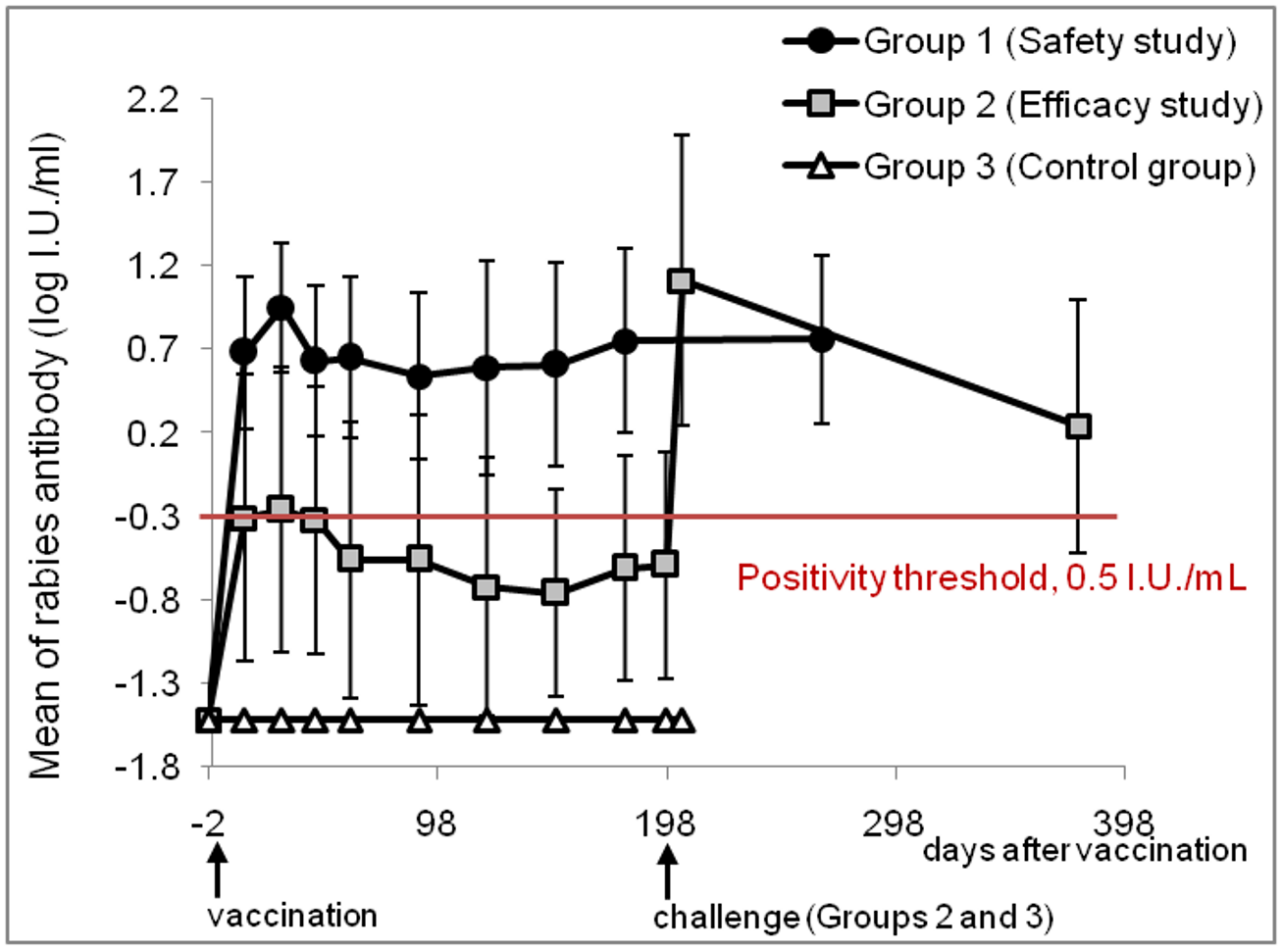
Rabies neutralising antibody (mean log IU/ml) kinetics of safety, efficacy, and control groups. On D0, the ferret badgers in Group 1 received a 10^9^/dose of SAG2, Group 2 received a 10^8^/dose of SAG2, and Group 3 received PBS (non-vaccinated). On D198, both Groups 2 and 3 were challenged with the Taiwan ferret badger rabies virus. Serum samples were measured with the fluorescent antibody virus neutralisation (FAVN) test, and titres are expressed through international units per ml (IU/ml). The symbols (circle, square, and triangle) represent means and standard deviations (SD).

During the observation period of 266 days, all the animals in Group 1 remained in good health without developing encephalitis or clinical signs suggestive of rabies. Rabies VNA at D-2, D14, D30, D45, D60, D90, D120, D180, and D266 detected in ferret badgers from Group 1 are displayed in Fig 1A and S1 Table, and the kinetics of mean Rabies VNA of Group 1 is presented in Fig 2. All the animals were seronegative before SAG2 administration. Notable seroconversions were demonstrated in all 10 animals on D14 (geometric mean = 4.80 IU/ml, SD = 5.63), and the highest levels of antibody titres within the group occurred on D30 (geometric mean = 8.97 IU/ml, SD = 10.27). Antibody titres measured on D45, D60, D90, D120, D150, D180 and D266 remained high (Figs 1A and 2), with titres not significantly different for each animal irrespective of the sampling date (p = 0.993).

#### Detection of rabies virus

RTCIT results of saliva swabs collected from Groups 1 and 3 on D-2 and from D1 to D7 inclusive were all negative. However, there were 50%, 20%, and 30% of positive SAG2 RNA detection rates in Group 1 by real-time RT-PCR from swabs collected on D1, D2 and D3 respectively, but no SAG2 RNA was detected from D4 to D7 (Table 1). Following euthanasia on D266, neither rabies virus antigens nor RNA could be detected by either FAT or real-time RT-PCR respectively (S2 Table). RTCIT was unable to isolate the virus from brain and salivary gland specimens collected from Group 1 animals after euthanasia on D266 (S2 Table).

**Table 1:** SAG2 virus excretion in the saliva of Formosan ferret badgers in the safety study.

	Test methods	Positive rate of detection, tested positive^a^/tested no.^b^ (%)							
		Pre-inoculation	Day1	Day2	Day3	Day4	Day5	Day6	Day7
**Group 1**	**RTCIT**	0/10 (0)	0/10 (0)	0/10 (0)	0/10 (0)	0/10 (0)	0/10 (0)	0/10 (0)	0/10 (0)
	**Real-time RT-PCR**	0/10 (0)	5/10 (50)	2/10 (20)	3/10 (30)	0/10 (0)	0/10 (0)	0/10 (0)	0/10 (0)
**Group 3**	**RTCIT**	0/8 (0)	0/8 (0)	0/8 (0)	0/8 (0)	0/8 (0)	0/8 (0)	0/8 (0)	0/8 (0)
	**Real-time RT-PCR**	0/8 (0)	0/8 (0)	0/8 (0)	0/8 (0)	0/8 (0)	0/8 (0)	0/8 (0)	0/8 (0)

RTCIT, rapid tissue culture infection test.

^a^ The total number of animals that were tested positive in the group.

^b^ The total number of animals in the group that were submitted to detection of SAG2 virus (with RTCIT) or RNA (with Real-time RT-PCR).

### Efficacy of SAG2 in Formosan ferret badgers

#### Clinical observation

**Fig 3:**
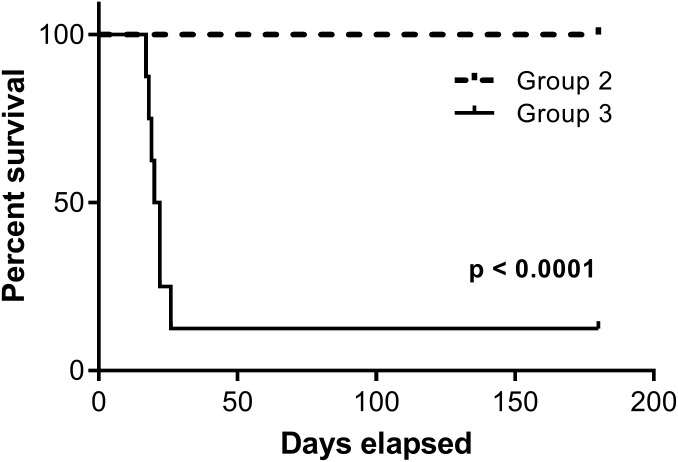
The survival curves of vaccinees and controls after rabies challenge. 198 day post-vaccination, vaccinees receiving SAG2 in 10^8^ per dose (Group 2) and controls in Group 3 were challenged with Taiwan ferret badger rabies virus in 10^3.2^ MICLD_50_. After 180-day observation, the statistic analysis showed a statistically significant difference on survival curves between Groups 2 and 3.

Before the challenge, all the vaccinated animals in Group 2 and the controls in Group 3 remained healthy from D1 to D198 inclusive, without any clinical signs evocative of rabies. After the challenge, 7 out of 8 control animals (Group 3) succumbed to rabies and died between 17 and 26 days post-challenge (20.57 days ± 3.05 days, Fig 3 and S2 Table). All 7 animals developed symptoms indicative of rabies before compassionate euthanasia. None of the 15 vaccinated animals from Group 2 died or showed any clinical signs evocative of rabies during the 180-day post-challenge observation period (Fig 3). The statistic analysis indicated a statistically significant difference on survival curves between Groups 2 and 3 after challenge (p < 0.0001, log rank test).

#### Rabies virus neutralising antibodies

Fig 2 shows the kinetics of rabies virus neutralising antibodies for Groups 2 and 3, while Fig 1B and 1C and S1 Table report the detailed titre for each animal in Groups 2 and 3. All the ferret badgers in Groups 2 and 3 were negative for rabies antibodies before vaccination. After vaccination, all the control ferret badgers (Group 3) remained negative for rabies VNA at all the blood sampling time points (Fig 1C and S1 Table). In contrast, low positive antibody responses were recorded for Group 2; VNAs were detected on D14 (geometric mean = 0.49 IU/ml, SD = 2.60) ranging from 0.03 to 8.69 IU/ml, but only 8 out of 15 ferret badgers presented a rabies neutralising antibody level that exceeded the positivity threshold (Fig 1B and S1 Table). Antibody titres peaked on D30 (geometric mean = 0.55 IU/ml, SD = 4.11), with 10 out of 15 animals having titres above 0.5 IU/ml. Subsequently, antibody titres declined slightly on D45 (geometric mean = 0.48 IU/ml, SD = 19.2) and D60 (geometric mean = 0.28 IU/ml, SD = 2.43) and then remained at levels similar to those on D60 until the challenge day (D198, geometric mean = 0.26 IU/ml, SD = 1.14), when only 6 out of 15 ferret badgers still had positive VNA titres (Figs 1B and 2; S1 Table). There was no significant difference in VNA titres from D14 to D198 using the Kruskal-Wallis test (p = 0.6444, Fig 1B). Nevertheless, 7 days post-challenge—in contrast to the completely negative VNA titres found in Group 3—positive responses were found in 13/15 vaccinees, and the geometric mean VNA reached 12.98 IU/ml (± 29.43, Figs 1B and 2). This rabies antibody response significantly increased between D198 and D205, suggesting a significant anamnestic response in 13 out of 15 vaccinated ferret badgers (p < 0.0001, Wilcoxon signed-rank test, Fig 1B). On D378, the antibody titres declined significantly (geometric mean = 1.73 IU/ml, SD = 4.62; p < 0.0001, Fig 1B) compared with values on D205, and 12/15 animals remained seropositive with titres still higher than those on D198 (p < 0.0001, Wilcoxon signed-rank test, Fig 1B).

#### Detection of rabies virus

After euthanising the diseased control animals from Group 3 and all the survivors from Groups 2 and 3 on D378, the FAT and RTCIT were positive for the 7 ferret badgers having succumbed to rabies in Group 3 (Nos. 26–32, S2 Table), and negative for the other 15 animals in Groups 2 (Nos. 11–25) and 3 (No. 33) (S2 Table).

## Discussion

Since the discovery in 2013 of rabies in ferret badgers, the Taiwanese government has decided to eliminate the virus nationwide to secure public health. This study includes two experimentations to assess on one hand, the safety, and on the other, the immunogenicity and efficacy of the SAG2 vaccine on ferret badgers. The key criteria for selecting a candidate vaccine are the safety for target and non-target species, and the efficacy of the vaccine strain according to WHO requirements [19]. In Europe, where more than 20 million SAG2 baits have been used, no safety issues have been reported in multiple target and non-target species, and several countries have been declared free of rabies after only a few years of oral vaccination campaigns with this vaccine [16].

The safety of SAG2 was assessed by directly instilling into the mouth of ferret badgers a dose 10 times that of the recommended dose (i.e. 10^9^ TCID_50_ per animal). All the animals in Group 1 remained perfectly healthy and did not show any symptoms up to 266 days post-instillation. In addition, no other infections were detected during the observation period. After euthanasia, no viral antigens, RNA or any replication-competent SAG2 virus were found in the animals’ brains or salivary glands. These findings are very similar to those of previous studies on other target and non-target animals, especially foxes and domestic dogs and cats, each administered with more than 10^9^ TCID_50_ and kept under observation for over 6 months [23, 31]. The rabies virus is generally transmitted through saliva; after oral vaccination, biting animals might transmit the vaccine virus accidentally. Although SAG2 RNA was detected on Days 1, 2 and 3 post-instillation at low levels that gradually declined (50%, 20% and 30%, respectively), no infective virus was detected on any of the saliva swabs from ferret badgers examined during this study. Cases of SAG2 virus recovery from oral swabs have only been documented in genets given a dose of 10^9^ TCID_50_ per animal on Day 1 [32], 3 jackals given a dose of 10^7.5^ TCID_50_ per animal on Day 3 after vaccination [24], and one dog (out of 19) one hour after bait consumption (vaccine dose of 10^8.3^TCID_50_ per animal) [33], and all with very low quantities recovered [24, 32, 33]. Furthermore, dogs have been shown to allow local replication of the SAG2 virus in the oral cavity with a peak on Day 3 (verified by testing mRNA and positive-stranded RNA of SAG2), but this cleared up quickly and no more virus was excreted [33]. In our study, as no infective SAG2 virus was isolated from any oral swab (total of 70 samples), and although positive detections of SAG2 RNA occurred once, followed by a decline and even disappearance of RNA detection, it could be deduced that the brief period of SAG2 RNA detection in this study is the residue of vaccine application, and not the result of local replication in the mouth. The persistence of the SAG2 virus in the oral cavity of Formosan ferret badgers is therefore considered null, as shown in most previous studies for target and non-target animals [16].

A vaccine candidate should be immunogenic for target animals, which is why the rabies VNA titres of vaccinated ferret badgers were measured. The antibody titres measured in animals from Group 3 were all 0.03 IU/ml, which corresponds to the baseline signal of non-vaccinated animals. An antibody titre of 0.5 IU/ml is currently considered to be the positivity threshold [34]. Eight and 10 of the 15 ferret badgers in Group 2 developed positive rabies VNA levels 14 and 30 days after vaccination respectively, and only 6 of these animals remained positive up to and including D198. Only two of the ferret badgers (Nos. 13 and 25) had a titre of 0.03 IU/ml after vaccination but before the challenge, a level similar to those of the control group. Furthermore, except on D30 when the mean antibody titre for Group 2 was greater than the positivity threshold, all the other mean antibody titres were less than 0.5 IU/ml before the challenge on D198. Compared to other animal species vaccinated with SAG2, ferret badgers seemed to be more refractory to neutralising antibody production elicited by the vaccine; the administration of a SAG2 vaccine bait of 10^8.15^ TCID_50_ to raccoon dogs (n = 29) or a SAG2 virus suspension of 10^8.3^ TCID_50_ instilled in red foxes (n = 8) led to 100% of the animals developing VNA titres greater than or equal to 0.5 IU/ml on D201 or D182 post-vaccination respectively [21, 27]. Rabies antibodies measured in raccoon dogs even ranged from 3.5 to 31.6 IU/ml 201 days after vaccination [21]. In contrast, lower antibody titres or limited seroconversion rates induced by SAG2 have been demonstrated in dogs (5 out of 9 dogs vaccinated with 10^8.5^ TCID_50_ seroconverted), skunks (2 out of 5 skunks vaccinated with 10^9^ TCID_50_ seroconverted), raccoons (3 out of 5 raccoons vaccinated with 10^9^ TCID_50_ seroconverted) and jackals (2 out of 3 jackals vaccinated with 10^7.5^ TCID_50_ seroconverted) [20, 24, 35]. In ferret badgers receiving one shot of 10^9^ TCID_50_ (Group 1), a significant seroconversion was observed in all animals at any serum sampling time from D14 to D266, suggesting a dose-dependent relationship between SAG2 application and VNA induction in ferret badgers, and further indicating lack of replication because if the virus replicated this would negate differences in the initial dose. Although antibody titres from the safety group are really high and a dose of 10^9^ TCID_50_ could be considered for natural immunization; however, from our results, the dose of 10^8^ TCID_50_ is sufficient to provide competent protection. Moreover, the most commonly used dose of SAG2 in wildlife in the field is 10^8^ TCID_50_ per dose [16], and this dose is also the formula currently in the market. In addition, a formula of 10^9^ TCID_50_ costs more for production and could not practically be applied in large-scale field distribution.

To ensure that SAG2 could provide sufficient protection for the ferret badger population, its efficacy was assessed by a challenge using a field rabies virus isolate (RABV-TWFB) on day 198 post-vaccination. Despite the low antibody titres elicited in ferret badgers immunised with the recommended dose (10^8^ TCID_50_), the efficacy is satisfactory because 100% protection was obtained in all 15 vaccinees, whereas 7 out of 8 unvaccinated control animals succumbed to rabies. The vaccinated animals remained healthy and rabies virus diagnosis was negative, demonstrating the absence of infection and full protection after oral vaccination. In contrast, the rabies virus was detected in the brain material of 7 control animals that died after being challenged. The one ferret badger that survived the challenge (No. 33) showed a slight increase in antibody titre on the day of euthanasia (0.31 IU/ml) and was negative for rabies diagnosis. The mean survival duration (20 days) was comparable to that reported in a study using nearly the same challenge dose (10^3.6^ LD_50_) for raccoon dogs (17 days) [21], and both met the requirements of European Pharmacopoeia monograph No. 0746 and U.S. Code of Federal Regulations (control animals should die within 90 days after being challenged) [36, 37]. In previous studies assessing the efficacy of SAG2 in different species, a 100% mortality rate was not systematically achieved in the control group. This was the case, for example, when assessing arctic foxes and red foxes: 3 out of 4 animals died due to rabies in the first study, and 7 out of 8 died in the second one [26, 38]. Although not all the control animals were killed by the rabies challenge, a 87.5% mortality rate is close to the requirements of European Pharmacopoeia monograph No. 0746 (a mortality of at least 90% is required in a control group of at least 10 animals) and those of the U.S. Code of Federal Regulations (80% of mortality in controls) [36, 37], and moreover, the statistical analysis showed a statistically significant difference on survival curves between vaccinees and controls after challenge.

On the day of the viral challenge, more than half of the vaccinees were still seronegative (9/15). One week after the rabies challenge, a very high and significant anamnestic response was recorded (13/15 were seropositive with high antibody levels), but all the control animals remained at the basal and negative VNA level. The two ferret badgers (Nos. 13 and 25) that did not seroconvert showed a slight increase in antibodies 7 days after the challenge (0.19 IU/ml and 0.14 IU/ml respectively). Both resisted the challenge and remained healthy, confirming the fact that protection against rabies can occur in the vaccinees despite a lack of detectable antibodies before rabies challenge [20]. After challenge, there was an antibody increase in SAG2-immunised ferret badgers of around 50-fold, which is much higher than any recorded increase in arctic foxes, red foxes, raccoons, skunks or jackals [24, 26, 35, 39], even though the seroconversion induced by SAG2 in ferret badgers was not as remarkable as that in raccoon dogs and red foxes. A study of the oral vaccination of Chinese ferret badgers with SRV9 (derived from SAD, an origin shared by SAG2) and CAV-2-E3D-RGP (canine adenovirus harbouring rabies virus glycoprotein) in 10^7.5^ plaque-forming units respectively revealed that 16 and 17 out of 20 animals developed positive antibody responses on Day 21, and mean titres were 1–1.6 IU/ml [40], higher than in this study; hence, ferret badgers do not innately generate low levels of rabies VNA, but a particular immunity activated by SAG2 in ferret badgers and characterised by satisfactory protection independent of humoral immunity induced excellent efficacy. Besides SAG2, this kind of live oral vaccine effect on vaccinated animals with a low seroconversion rate but high protection rate has already been fully documented for another live oral rabies vaccine, the vaccinia-recombinant rabies vaccine [41, 42]. Therefore, for both dogs and ferret badgers, cell immunity might play an important role in the protection mechanism induced by a live vaccine [27].

In this study, the safety and efficacy of SAG2 for ferret badgers have both been fully investigated and demonstrated. No adverse reactions were detected after the oral administration of SAG2 vaccine at 10 times the field dose. The vaccine induced full protection in vaccinated animals after a virus challenge that killed 87.5% controls. However, before distributing SAG2 vaccine in the field, additional investigations have to be made to assess the vaccine’s safety for sympatric species (e.g. gem-faced civets) and to evaluate the immune effects after ferret badgers have consumed vaccine baits in the field in the framework of a well-controlled pilot project. The low seroconversion rate induced by SAG2 in ferret badgers might confuse assessment of the appropriate immune coverage following vaccination in the field; besides VNA titration, other protection indexes or bio-marker determination should be considered and applied as is currently the case in Europe [43].

## Supporting information

S1 Table All data of rabies virus neutralising antibody titres (IU/ml) of Formosan ferret badgers in this study. (XLSX)

S2 Table All data of responses to rabies challenge and rabies diagnosis in this study. (XLSX)

S3 Table ARRIVE guidelines checklist. (PDF)
